# Parvovirus B19 Infection Presenting as Acute Anemia and Polyarthropathy

**DOI:** 10.7759/cureus.104656

**Published:** 2026-03-04

**Authors:** Peter Young, Omer E Beaird

**Affiliations:** 1 Internal Medicine/Division of General Internal Medicine and Health Services Research, David Geffen School of Medicine, University of California Los Angeles, Los Angeles, USA; 2 Infectious Disease, University of California Los Angeles, Los Angeles, USA

**Keywords:** acquired aplastic anemia, parvovirus b-19, polyarthropathy, viral arthritis, viral infection-associated aplastic anemia

## Abstract

Human parvovirus B19 is a common viral pathogen in humans with increasing prevalence in the United States. While it is classically taught as an important cause of transient aplastic crisis in patients with sickle cell disease, it is usually a mild, self-limited illness in healthy hosts. We report a 46-year-old woman who presented with normocytic anemia, rash, and arthropathy after a flu-like syndrome and was found to have acute parvovirus B19 infection. She improved with supportive care. This case highlights the varied manifestations of parvovirus B19 infection and the importance of recognizing it as a cause of acute arthropathy with rash and anemia.

## Introduction

Parvovirus B19 is a common viral pathogen in humans presenting with a biphasic illness, initially causing flu-like symptoms that can be followed by rash and arthropathy [1,2]. While infection is usually self-limited and mild, parvovirus can also cause severe illness in immunocompromised individuals or people with chronic hemolytic disorders [3]. Most healthy patients with parvovirus B19 do not seek medical care, but serologic sampling in the United States shows a rising prevalence of infection [4,5]. Clinicians who recognize parvovirus B19 as a cause of transient anemia and arthropathy can limit unnecessary testing and provide reassurance to patients presenting with acute symptoms.

## Case presentation

**Figure 1:**
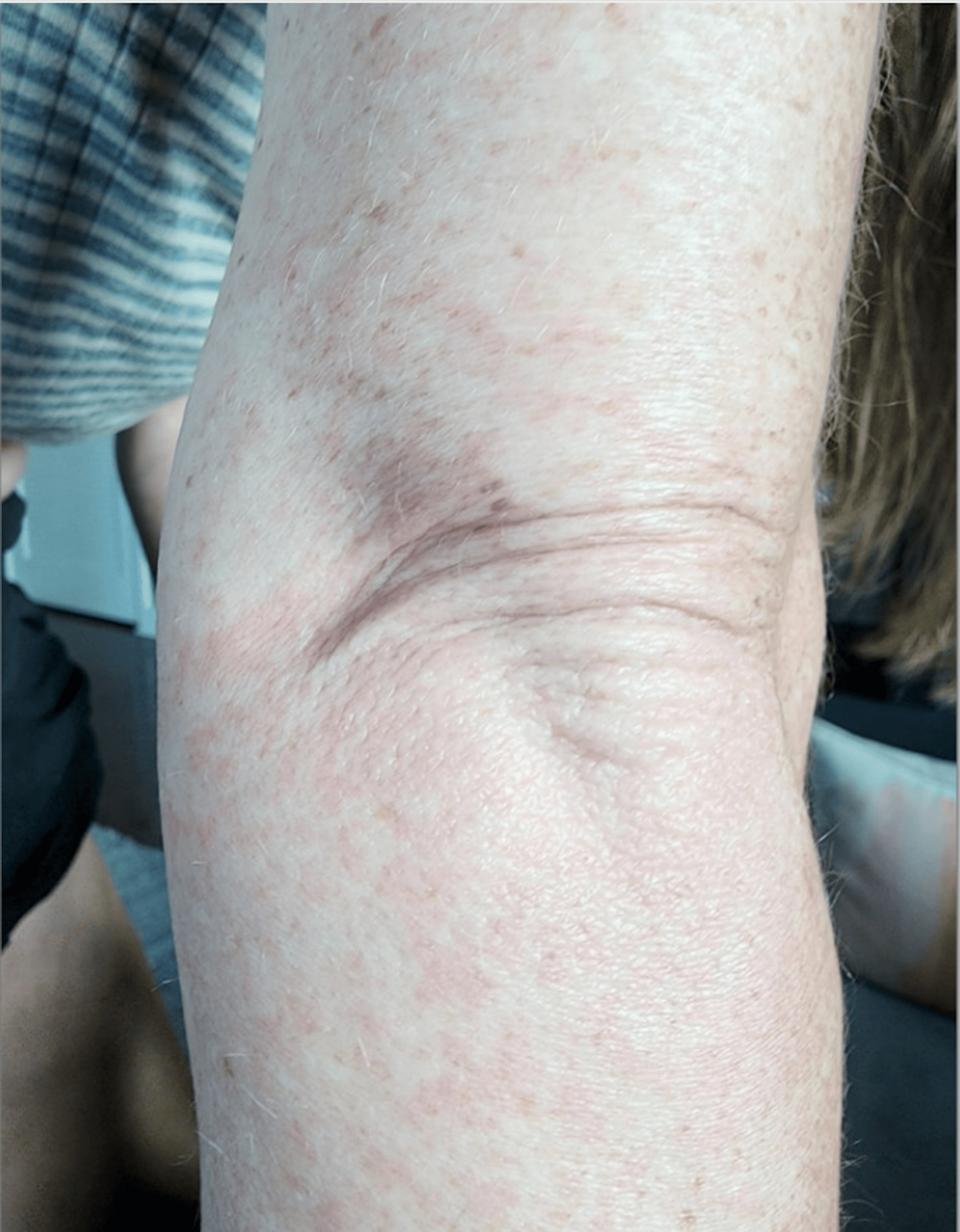
Erythematous Rash on Arm This image shows an erythematous and reticulate rash present on the patient’s arms

A 46-year-old woman with a history of gastrointestinal visceral myopathy and short-gut syndrome on total parenteral nutrition (TPN) presented with one week of rash, joint pain, arm swelling, headache, and malaise. Her rash was lacy, erythematous, and reticular. It had started on her arms before spreading to her legs and trunk (Figure 1). Two days after the rash appeared, she noted joint pain and stiffness in her knees, wrists, fingers, and elbows, along with back pain. She had mild joint swelling, particularly in her right elbow, wrist, and hand joints. She had a headache and mild lightheadedness, but no fevers, chills, or any other infectious symptoms. She had no history of prior rheumatologic disease. She initially presented to the emergency department for evaluation.

In the emergency room, she had a duplex ultrasound of her upper extremities, which was negative for any thrombosis. Labs were obtained, which showed a new normocytic anemia with hemoglobin of 9.6 and an increase in her liver function tests (LFTs), which were elevated at baseline due to parenteral nutrition-associated liver disease. Relevant labs are summarized below in Table 1 along with her baseline lab values from one month prior to presentation.

**Table 1:** Summary of Relevant Lab Results from Emergency Room Visit Lab results from the emergency room evaluation. The patient's baseline lab results are included for comparison. Notable findings include a new normocytic anemia along with a mild increase in her baseline elevated LFTs. mmol/L: millimoles per liter, mg/dL: milligrams per deciliter, U/L: units per liter, g/dL: grams per deciliter.

Lab Test	Reference Range	Lab Value (In Emergency Room)	Lab Value (At Baseline)
Sodium	135 - 146 mmol/L	139	139
Potassium	3.6 - 5.3 mmol/L	4.4	4.2
Chloride	96 - 106 mmol/L	105	105
Total CO2	20 - 30 mmol/L	24	25
Anion Gap	8 - 19 mmol/L	10	9
Glucose	65 - 99 mg/dL	101	99
Creatinine	0.60 - 1.30 mg/dL	0.71	0.65
Urea Nitrogen	7 - 22 mg/dL	25	23
Calcium	8.6 - 10.4 mg/dL	9.2	9.4
Total Protein	6.1 - 8.2 g/dL	6.5	6.5
Albumin	3.9 - 5.0 g/dL	3.9	3.9
Bilirubin, Total	0.1 - 1.2 mg/dL	0.8	0.88
Alkaline Phosphatase	37 - 113 U/L	662	481
Aspartate Aminotransferase (AST)	13 - 62 U/L	85	58
Alanine Aminotransferase (ALT)	8 - 70 U/L	116	63
Hemoglobin	11.6 - 15.0 g/dL	9.6	11.9
Hematocrit	35.5 - 44.9%	27.70%	35.30%
Mean Corpuscular Volume (MCV)	78.2 - 97.9 fL	92.3	93.9
Platelet Count	157 - 371 x 10^9^/L	157	145
Leukocytes	3.4 - 9.6 x 10^9^/L	5.7	4.1
Neutrophils	1.56 - 6.45 x10^9^/L	4.54	2.5
Lymphocytes	0.95 - 3.07 x10^9^/L	0.71	1.2

She was discharged and advised to follow up with her primary care provider (PCP). This visit occurred four days after she was treated in the emergency room. Further history revealed that one week prior to the rash and joint pain, she had experienced several days of low-grade fever, headache, rhinorrhea, and sore throat suggestive of an upper respiratory infection (URI). Her vitals in the office were unremarkable. The physical exam was notable for a faint erythematous reticulate maculopapular rash on her arms. A head, eyes, ears, nose, and throat (HEENT) examination was unremarkable, as was her cardiopulmonary exam. Joint exam did not reveal any redness or warmth but was notable for mild bogginess and discomfort with passive manipulation of her metacarpal phalangeal (MCP), proximal interphalangeal (PIP), and distal interphalangeal (DIP) joints bilaterally, as well as her hips and knees. Her central venous catheter for TPN administration was a tunneled right subclavian line, and its site was clean, dry, and intact.

She was sent for further lab testing, which is summarized below (Table 2). She had mild elevation of her erythrocyte sedimentation rate (ESR) and a normal C-reactive protein (CRP). Iron studies revealed an elevated ferritin (224 ng/mL). She did not have any evidence of hemolysis, with lactate dehydrogenase (LDH) of 176 U/L and a haptoglobin of 99 mg/dL, and no signs or symptoms to suggest recent blood loss. She had an elevated reticulocyte count of 4.79%, which was slightly below the expected bone marrow response for her degree of anemia. Nutritional deficiencies (B12, folate, and iron) were considered a cause of her anemia, but her labs did not support this. Given the biphasic nature of her symptoms following a URI, along with joint pains, rash, anemia, and LFT abnormalities, there was suspicion for a post-viral syndrome. She was previously vaccinated against mumps, measles, rubella, and hepatitis B with documented immunity and had no risk factors for exposure to human immunodeficiency virus (HIV) or hepatitis C. Serologic testing (Table 3) was sent for parvovirus B19, Epstein-Barr virus (EBV), and cytomegalovirus (CMV), which returned with elevated parvovirus B19 IgM antibodies, consistent with recent parvovirus infection.

**Table 2:** Labs From Emergency Room Follow-Up Visit Lab results obtained at the patient's follow-up appointment, four days after her emergency room visit. mm/h: millimeters per hour, mg/L: milligrams per liter, mcIU/L: milli-international units per liter, ng/mL: nanograms per milliliter, mcg/dL: micrograms per deciliter, pg/mL: picograms per milliliter

Lab Test	Reference Range	Lab Value
Erythrocyte Sedimentation Rate	2 - 20 mm/h	35
C-Reactive Protein	< 5.0 mg/L	< 3.0
Thyroid Stimulating Hormone (TSH)	0.3 - 4.7 mcIU/mL	1.6
Ferritin	8 - 180 ng/mL	224
Iron	41 - 179 mcg/dL	43
Iron Binding Capacity	262 - 502 mcg/dL	339
% Saturation	15 – 45%	13%
Folate, Serum	8.1 - 30.4 ng/mL	11.5
B12	254 - 1,060 pg/mL	1,383
Lactate Dehydrogenase	125 - 256 U/L	176
Haptoglobin	21 - 210 mg/dL	99
Reticulocyte Count	0-2.5%	4.79%

**Table 3:** Viral Antibody Testing Results are consistent with a recent parvovirus B19 infection based on the elevated parvovirus B19 IgM titers Ig: immunoglobulin, IU: international units

Lab Test	Reference Range	Lab Value
Parvovirus B19 IgG	< 0.90 IU	7.21
Parvovirus B19 IgM	< 0.89 IU	16.65
Ebstein-Barr Virus (EBV) IgG	-	Positive
Ebstein-Barr Virus (EBV) IgM	-	Negative
Ebstein-Barr Virus (EBV) Polymerase Chain Reaction (PCR)	-	Undetectable
Cytomegalovirus (CMV) IgG	-	Negative
Cytomegalovirus (CMV) IgM	-	Negative
Cytomegalovirus (CMV) Polymerase Chain Reaction (PCR)	-	Undetectable

Based on her symptoms, lab work, and positive IgM antibodies, the patient was diagnosed with acute parvovirus B19 and treated with supportive care. Given her TPN-associated liver disease and short-gut syndrome, oral non-steroidal anti-inflammatory drugs (NSAIDs) were avoided, but she had symptom relief with topical diclofenac. As shown in Table 4, her bone marrow response, as assessed by reticulocyte index, was lower than expected during recovery, which her providers hypothesized could be related to her underlying comorbidities. Her joint pain gradually improved over six weeks, and her anemia resolved after three months.

**Table 4:** Serial Blood Count Monitoring Following Initial Presentation This table shows her gradual recovery from acute anemia, with hemoglobin restored to her baseline value three months following her parvovirus B19 infection. *Reticulocyte Index: > 3: appropriate bone marrow response to anemia, 2-3: borderline response, <2: inadequate response

Lab Test	Reference Range	Lab Value at Primary Care Visit	Lab Value Two Weeks Later	Lab Value Three Months Later
Hemoglobin	11.6 - 15.0 g/dL	10.5	10.9	12.2
Hematocrit	35.5 - 44.9%	27.70%	32.6	36.9
Red Blood Cell Count	3.92 - 5.13 x10^12^/L	3.41%	3.39	4.03
Reticulocyte Count	0-2.5%	4.79%	2.67%	-
Reticulocyte Index	* See below	2.2	1.5	-

## Discussion

Parvovirus B19 is the only human pathogen in the Parvoviridae family capable of causing varied clinical presentations and syndromes in children and adults [6]. Most cases of parvovirus B19 are mild and self-limited, though presentation varies depending on host factors [3]. In children, parvovirus B19 infection classically presents as erythema infectiosum, also referred to as “fifth disease,” which begins with flu-like symptoms of fever, coryza, headache, nausea, and diarrhea [6]. An erythematous malar rash with circumoral pallor, often called a “slapped cheek" rash, may appear afterwards and can spread to involve the trunk and extremities (Bloise). In adults, the classical “slapped cheek” rash is less common. Instead, adult patients often experience a symmetric polyarthropathy with joint swelling, which can be mistaken for Lyme disease or rheumatoid arthritis [7]. In patients with chronic hemolytic diseases such as sickle cell disease, infection can cause a serious transient aplastic crisis [8]. In immunosuppressed patients, it can cause pure red blood cell aplasia, as well as chronic and recurrent infections [7]. Infection during pregnancy can lead to miscarriage, fetal demise, and hydrops fetalis [9].

Parvovirus commonly presents as a biphasic illness [2]. After exposure, flu-like symptoms develop within four to 14 days, followed by a second phase of illness with rash and arthralgias, though there is significant heterogeneity in the timing of these phases, which can sometimes appear simultaneously [10]. In children, the presenting rash classically starts around the face as malar erythema with circumoral pallor before spreading to the trunk and limbs, while adults may display a lacy, reticulate, erythematous rash that starts on the limbs and often does not involve the face at all [10]. The rash from parvovirus is immune-mediated and usually appears after the resolution of viremia and initial prodromal symptoms [3].

Arthritis and arthralgias, as described in this case, affect up to 60% of adults but less than 10% of children and have a preponderance for women [1,10,11]. The typical presentation is symmetric pain, swelling, and stiffness, which most commonly affects the metacarpophalangeal joints but can also include the knees, wrists, and ankles [1,11]. Symptoms are related to immune activation, and parvovirus B19 has been found in the synovial fluid of affected joints [11]. The arthropathy typically lasts for one to three weeks, but 20% of women can have prolonged symptoms lasting several months [11]. Symptoms can be managed with NSAIDs and supportive care. 

As in this patient, anemia can be a common complication of parvovirus infection, though it is usually of minimal clinical significance in immunocompetent individuals [8]. Parvovirus B19 induces apoptosis of erythroid-lineage cells, which causes bone marrow failure [8]. Most patients experience a reduction in reticulocytes lasting 2-5 days [1]. In patients with hemolytic diseases, notably sickle cell disease, parvovirus B19 can cause a transient aplastic crisis, which can lead to severe, sometimes life-threatening anemia [12]. The patient may require transfusion and supportive care for one to two weeks until viremia clears, after which erythrocyte production usually returns to normal [12]. This patient’s reticulocyte count of 4.79% was not consistent with bone marrow failure, but this bloodwork was drawn two weeks after suspected infection and may have represented some recovery of erythroid precursor cells.

Infection can be more severe in immunocompromised individuals, such as those who have undergone solid organ or hematopoietic stem cell transplantation or people with HIV/AIDS. Fever, rash, and arthralgia are less common, but anemia is almost universally present and often profound due to viral replication within erythroid progenitor cells, resulting in lysis and leading to severe and chronic pure red cell aplasia with associated reticulocytopenia. Infection can also present with pancytopenia. Infection can be persistent, and control ultimately requires effective humoral immunity. Treatment in this population typically involves reduction of immunosuppression if possible, along with high-dose intravenous immunoglobulin (IVIG), which is generally effective, but up to 28% of patients will have a relapse [13].

Transmission of parvovirus B19 occurs via respiratory droplets; transmission peaks during late winter through early summer [7]. Exposure is common during childhood, with 60-60% of young adults having been infected, and antibody positivity is approximately 90% by old age [7]. There may be periodic epidemics of parvovirus B19 with increased community transmission. While there is no program of routine surveillance and infection is not a notifiable condition in the United States, recent reports suggest an increase in disease activity [4].

## Conclusions

Our case demonstrates the importance of recognizing parvovirus B19, which can have varied presentations, especially in adults. Parvovirus B19 is most often thought of in the context of the severe complications it can cause for patients with hemolytic disorders or immunosuppression, but most infections are mild and self-limited. The triad of rash, arthralgias, and joint swelling, particularly when preceded by a flu-like illness, should prompt consideration of parvovirus B19. The acute timeline of symptoms differentiates post-infectious syndromes from underlying rheumatologic disease. Supportive care is the mainstay of treatment, and further diagnostic testing is usually not indicated for immunocompetent and otherwise healthy individuals. In patients with severe immunocompromise, a diagnosis should be considered if a patient presents with isolated anemia and treatment includes reduction of immunosuppression and IVIG. While the symptoms can be mistaken for other infections or rheumatologic conditions, early identification can mitigate the need for excessive workup and provide reassurance to patients.
